# Key Factors Affecting Mathematics Teachers’ Well-Being and Stress Levels: An Extended Engagement Theory

**DOI:** 10.3390/ijerph20010548

**Published:** 2022-12-29

**Authors:** Xin Jian, Tommy Tanu Wijaya, Qingchun Yu

**Affiliations:** 1 School of Mathematics and Statistics, Guangxi Normal University, Guilin 541006, China; 2 School of Mathematical Sciences, Beijing Normal University, Beijing 100875, China

**Keywords:** engagement, mathematics teacher, well-being, TPMK, stress

## Abstract

The mathematics teachers’ profession often has many challenges. It also occupies important positions at the K-12 education level, in which mathematics knowledge is the basis of all scientific fields. This tends to cause high-stress levels and a negative effect on well-being. Mathematics teachers’ well-being has been less examined, and therefore this study aims to determine the factors affecting mathematics teachers’ well-being and stress levels. The 210 data points collected from Chinese mathematics teachers using a web-based questionnaire were analyzed for reliability and validity, then model fit and SEM were applied for model validation after removing 3 invalid data points and incomplete responses. The results showed that behavioral and cognitive engagements significantly affect teachers’ well-being, while the affective engagement was insignificant. The TPMK was the strongest significant predictor that had a positive impact on improving well-being and reducing stress levels. In addition, the stress level of mathematics teachers was influenced by gender and age. Finally, it was proven that teachers’ well-being significantly reduced stress levels. This study’s implication was to provide information on how to reduce stress levels.

## 1. Introduction

The burden on mathematics teachers increases with time. To become competitive teachers in the 21st century, there is a need to master both mathematical as well as good pedagogical and technological knowledge [1,2]. This is the reason the Chinese government issues programs to improve teachers’ competence every year [3,4]. Education reform in the country was performed to enhance mathematics learning quality and the teaching standard [1]. After the COVID-19 pandemic, the Chinese government had a program to support sustainable learning from 2020 to 2025 [5]. It was believed that mathematics education in the future can be implemented both in the classroom and at home or around the students’ environment [6]. Therefore, the government has taken various steps to improve the quality of sustainable education in the country [7,8,9].

It is important to note that several tasks have to be performed to achieve the government’s program for improving and supporting sustainable education [10,11]. Multiple tasks can cause the teachers to be under constant pressure from school inspectors [12,13]. During the summer and winter holidays, there are many training courses on learning media or dynamic mathematics software for improving technological teaching skills [14,15,16]. Additionally, teachers have to prepare lesson plans during holidays for the next semester. This shows that they function as educators in the classroom and as observers, planners, facilitators, and role models for the community. In general, teaching has several great responsibilities, similar to other important professions. Hence, there is a need to consider mathematics teachers’ well-being and stress levels, related to their job performance, particularly while designing learning strategies and creativity in developing students’ practice questions and exams. Based on this background, it is suggested that the factors influencing teachers’ well-being and stress should be studied to reduce their stress levels.

Several studies examined the factors associated with stress levels in the education sector. Sood [17] examined the relationship between resilience, psychological well-being, and stress levels, while Yusli [18] concluded that restorativeness is related to stress levels. According to Park [19], coping strategies are important factors affecting stress. However, limited studies examined the relationship between engagement and stress levels, specifically in mathematics teachers [20].

Several theories suggested that stress levels are related to engagement. Based on the engagement theory proposed by Frederick [21], well-being is closely related to cognitive, affective, and behavioral engagements. The relationships among these three elements affect individual activities and resources for self-development. Therefore, engagement theory can be used as a strong theoretical basis for determining factors affecting an individual’s well-being and stress levels.

Other studies found that poor well-being leads to stress [22,23,24]. Meanwhile, high stress and low well-being usually cause poor performance and excessive fatigue [25,26,27]. It was observed in Malaysia that 90% of teachers consider resignation due to high-stress levels [28]. Another study discovered that stress levels decrease teaching quality, job satisfaction [29,30], self-confidence, and creativity [31], thereby affecting education in general [32,33,34]. Therefore, it is important to pay attention to stress levels in the teaching profession.

Based on the described background, this current study aims to analyze the factors related to mathematics teachers’ well-being and stress levels, which were developed from engagement theory combined with the knowledge of pedagogical mathematical technology. The results served as solutions to reduce teachers’ stress levels while improving their well-being in China. Furthermore, the differences between the construct variables based on demographic information, namely gender and age, were also examined. To achieve the objectives, data were collected using a questionnaire and the hypothesis was tested with a structural equations modeling (SEM) approach. Presently, the SEM has been the most trusted approach for analyzing the relationship between constructs [26,35,36]. Furthermore, this study is structured as follows.

The next section is a literature review that explains the stress levels, well-being, engagement theory, and TMPK, followed by the initial hypothesis preparation. In the third section, the methodology for preparing the instruments, data collection, and processing is explained. The fourth section entails the result and discussion, while the fifth consists of the theoretical and practical implications, and the last consists of the conclusion.

## 2. Literature Review

### 2.1. Mathematics Teachers and Stress Levels

In China, every learning activity and objective, plus mathematics textbooks, are determined through the standard curriculum issued by the Ministry of Education [37,38,39]. Furthermore, students’ mathematical abilities to be improved, as well as the educational approaches and technologies that need to be incorporated during teaching and learning, are suggested by the Ministry of Education [40,41]. All school principals and supervisory agencies are responsible for monitoring the teaching and learning development activities appropriate based on the national standard curriculum [42]. Education units in the regions regularly conduct training to improve learning quality. They also conducted regular training sessions outside of school hours on the application of ICT-based learning media, approaches, and new models in teaching and learning activities, which all teachers are required to attend [43,44,45]. Additionally, all teachers are advised to obtain a quality certification, which is issuable after: (1) having sufficient teaching experience, (2) being homeroom teachers, (3) attending regular training, (4) having teaching achievements in schools, (5) participating in learning design competitions, and (6) publishing scientific articles as added value [46,47]. This is related to their affective engagement, in which continuous effort is made to improve teaching performance and adopt new learning media, indicating that non-teaching goals and activities can physically exhaust and increase teachers’ stress.

It has been proven that Chinese teachers are under pressure in various ways [48,49]. This is because they are both responsible for classroom teaching as well as involved in various school activities as administrators. First, regarding workload, teachers often perform activities, such as meeting and communicating with parents about their children’s learning, in addition to daily lesson preparation and homework assessment [50]. This typically increases their workload and causes them to experience high-stress levels [51]. Second, in terms of academic achievement, many private secondary schools still prioritize students’ achievement due to the teaching ideology entrenched for years, despite the efforts made by the government to implement quality education reforms. This eventually places the front-line teachers under great stress. Finally, concerning role-taking, teachers in this country often perform multiple tasks. In addition to their teaching and educational responsibilities in the classroom, teachers are often required to act as administrators in organizing student activities and classroom management. These multiple roles cause them to feel overwhelmed and stressed.

Teachers, as well as education quality and development, are affected by their well-being [52,53]. Therefore, the social consensus is that professional welfare should be improved. Respecting teachers and improving their professional well-being are closely associated with teaching quality and education development. In other words, the teachers’ welfare is related to students’ development, meaning only those with high well-being levels can show enthusiasm in daily teaching activities and positively coordinate students to become outstanding.

### 2.2. Theoretical Foundation and Initial Hypothesis Development

#### 2.2.1. Theory of Engagement

Previous studies showed that engagement has a significant relationship with human well-being [54,55]. At secondary schools, mathematics teachers tend to have a high risk of being exposed to stress. Therefore, engagement is needed to reduce stress levels and maintain well-being. It also helps in maintaining teachers’ enthusiasm and motivation to teach [56]. In a previous study, engagement was considered less defined and theorized when treated as an extraneous construct [57,58]. Conceptually, it is the quantity and quality of mental resources focused on an object, emotion, and required behavior. Based on the subject matter, engagement is defined as a manifestation of mathematics teachers’ motivation, as measured by cognitive and affective behavior, related to vigorous, purposeful, and persistent action in the face of difficulties associated with the teaching profession. The theory is divided into three dimensions, namely behavioral, cognitive, and affective engagements [21].

Behavioral engagement is the individual’s willingness, intensity, and concentration to achieve a target and stay focused on the final goal [59,60]. This attitude also signifies perseverance and determination when facing difficulties. Teachers’ behavioral engagement in this current study is to: (1) prepare teaching materials, (2) efficiently teach mathematics to students, (3) persist when encountering difficulties in teaching activities, and (4) have a strong determination to improve their abilities regarding the subject. Some studies supported the relationship between behavioral engagement and well-being [61,62]. Therefore, the initial prediction is that behavioral engagement is related to well-being.

Affective engagement is about individuals’ emotions concerning what they are doing [63]. Another source explained this phenomenon as the quality of emotional reactions while performing an activity, as indicated by an individual’s enthusiasm, enjoyment, pleasure, and satisfaction [60,64]. In this context, this engagement refers to the feelings related to the teaching process. An indication of interest in teaching and learning activities includes enjoying teaching mathematics, meeting students, etc. Some studies showed that affective engagement is the strongest factor influencing subjective well-being [61,65]. Therefore, it is predicted that there is a relationship between affective engagement and mathematics teachers’ well-being.

Cognitive engagement is an individual’s investment in learning, characterized by a desire to take on more challenging tasks [64,66]. Usually, people with cognitive engagement have good coping strategies to deal with failure [67]. This engagement refers to the attitude of alertness, focus, concentration, participation, and willingness to exceed their abilities [64]. In this context, mathematics teachers’ cognitive engagement is a sense of always wanting to learn and develop teaching abilities. It also involves learning to improve teaching performance, developing or adopting learning models and media to enhance students’ mathematical abilities, and a sense of freedom from the burden of teaching [66]. Previous studies discovered that cognitive engagement is related to individuals’ abilities and influences well-being [68,69]. Therefore, it is hypothesized that cognitive engagement is related to well-being.

#### 2.2.2. Technological Pedagogical Mathematical Knowledge (TPMK)

TPMK was developed by Shulman [70] from the TPACK framework to develop good teaching methods and components to achieve 21st century teaching performance. According to Shulman [70], learning and teaching certain content with a pedagogical approach is less effective. Therefore, in line with technological developments, TPACK is recognized as effective technology integration in teaching and learning activities [71,72,73]. In this context, content knowledge is more related to mathematics, and hence it can be described as mathematical knowledge.

The difference between teachers’ performances when teaching with good and low TPACK has been observed. Other studies have also shown a relationship between TPACK and stress levels [74]. It was discovered that learning technology in the classroom increases students’ interest and motivation. Mathematics teachers with good TPMK can develop technology and combine ICT-based learning media in line with the learning model and content studied by students. Asamoah [75] stated that at the initial stage, teachers find it difficult to teach by integrating technology, but after mastering the system, they felt less stressed compared to when teaching with the face-to-face method. From this background, it was concluded that mathematics teachers’ stress levels and well-being are related to their TPMK.

#### 2.2.3. Teachers’ Stress Levels

Stress is a natural response that is inseparable from human life [76,77]. It is a physical and emotional response to the dynamics of life. Besides, it is also an intense and non-specific response to changes or challenges caused by both internal human and external environmental factors.

Stress is a hypothetical construct representing an individual’s state of balance in response to environmental and work demands [78,79]. Another definition of stress is the inappropriate bodily response to the demands of the surrounding environment [80,81]. In this context, mathematics teachers’ stress is described as an unpleasant emotional experience, frustration, anxiety, anger, and depression towards their job. Previous studies extensively analyzed teachers’ stress, which directly affects teaching and learning activities [25,49,82]. It was observed that stress affects teachers’ physical and mental health [83,84]. Excessive stressful conditions can also cause teachers’ depression and reduce teaching performance, which interferes with sustainable education. In other words, teachers’ stress levels affect students’ learning outcomes. From the beginning, the American government has focused on dealing with stress to prevent teachers’ attrition costs [85]. Even though many studies have examined stress levels in individuals, they are still on a small scale and not from the education field [86]. This showed the need for supporting studies to analyze the stress effects in every field and work profession. Borg [87] stated that the teaching profession often has stressors from the work environment due to some factors, namely changes in learning methods and objectives, the national curriculum, assessment, and school organization. Based on the above results, there is a need for a continuous study to examine the effects associated with mathematics teachers’ stress levels and solutions to overcome them.

#### 2.2.4. Teachers’ Well-Being

Well-being is difficult to define and measure. There are several definitions of well-being, and one example is that of O’Sullivan et al. [88], who described it as a state of being psychologically healthy, positive, and encouraged. Specifically, it is healthy emotion regulation, namely high reappraisal emotion regulation, and low-stress levels [89,90,91]. According to some studies, well-being can be divided into objective and subjective. The objective well-being is measured by economic status, health, and relationships with other people, while the subjective includes experience, happiness, emotions, life goals, competencies, and a person’s abilities. Studies on well-being focused more on the workforce, while those in the education field are still very limited. It was discovered that the positivity of teachers and job satisfaction are influenced by individual well-being. Subjective well-being is believed to influence all domains in one’s life [92]. Furthermore, studies in the education field increased due to the positive psychology emergency in the 20th century. The literature demonstrated that a teacher’s well-being levels directly psychologically affect students and eventually influence their outcomes [93,94]. In North Korea, high well-being levels tend to improve the interaction between teachers and students [95].

Teachers’ well-being is essential to students’ learning environment and classroom practice. According to Byun [95], this phenomenon is affected by many factors; hence, empirical evidence is needed to examine these factors. This study focuses on developing models and predicts that well-being is affected by engagement and TPMK.

**Figure 1:**
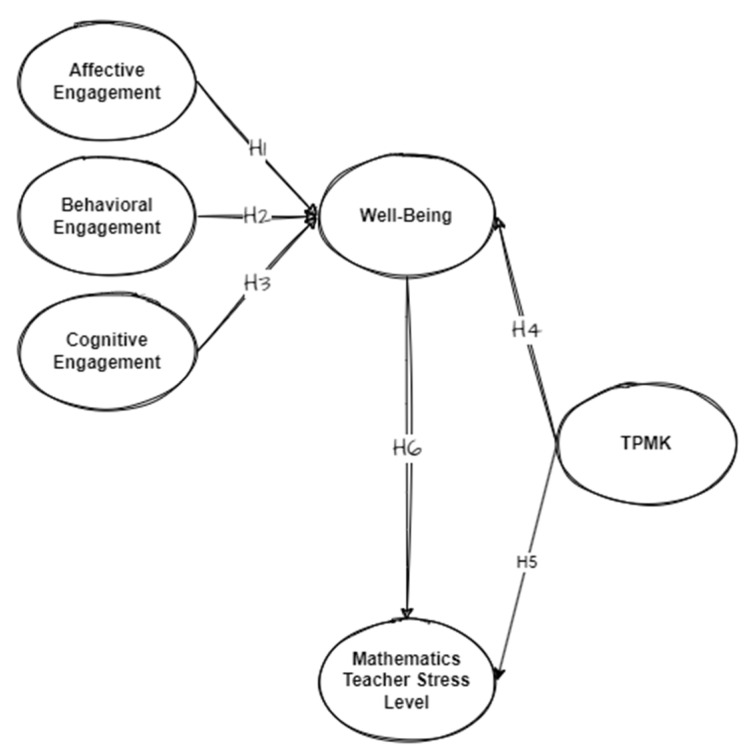
Study framework. Note: TPMK: Technological Pedagogical Mathematical Knowledge.

Based on the literature review, this study consists of four dependent variables, an intermediate variable, and two independent variables, with six initial hypotheses. The details are shown in Figure 1 and Table 1. Furthermore, several studies reported the differences in gender, age, and education level [96]. In this study, the levels of engagement, stress levels, and well-being were assumed to be influenced by gender and age. 

## 3. Methodology

A quantitative method was utilized by designing a questionnaire to test the initial analysis for determining the factor that has a significant effect on mathematics teachers’ stress levels and well-being. The first step was determining the variables and measurement items through a literature review. Furthermore, the questionnaire was adopted from previous studies and adjusted to suit this context. The initial data screening, demographic respondents, normality test, validity, reliability, and initial hypothesis testing in this study were assisted by SPSS 25 and Smart-PLS software 22.

### 3.1. Questionnaire Development

Questionnaire items were compiled based on the engagement theory [21], stress level [97], well-being [7,98], and TPMK [1,4]. The literature related to predictors was analyzed, while the appropriate questionnaire items to be adopted were also compared and determined. In the next stage, the measurement model design and questionnaire items were discussed with two doctors and professors who are experts in psychology, education, and mathematics education, as well as being experienced in investigating well-being and stress levels. The 6 variable constructs have 4 questionnaires, thereby amounting to 24 items. The questionnaire was prepared using a 5-point Likert scale ranging from strongly disagree (1) to strongly agree (5).

### 3.2. Study Sample and Data Collection Procedure

The sample employed consisted of mathematics teachers in Guangdong Province, China. Questionnaires were distributed using random sampling and the focus was the teachers at the junior and high-school levels. This is because mathematics material is still quite easy at the elementary level. Some are homeroom teachers and consider teaching other subjects, such as English or sciences. A total of 250 questionnaires were distributed using the *wenjuanxing* application, whose opening section explained that this study was conducted by Guangxi Normal University to determine the factors related to mathematics teachers’ stress levels. Furthermore, it explained that the respondents’ data were confidential and safe, and hence the teachers can participate voluntarily without coercion. The data collection was performed for three weeks from September to October 2022.

The initial analysis showed that 211 mathematics teachers voluntarily filled out the questionnaire, but only 207 completed and analyzed it. The basic information of respondents included 178 female and 29 male teachers. In total, 21, 91, 79, and 16 persons were aged 20–30, 31–40, 41–50, and above 50 years, respectively. Approximately 7 teachers had 1 year of teaching experience, while 15, 5, and 180 persons had 1–3, 3–5, and above 5 years, respectively. Moreover, 169 teachers had a bachelor’s degree and 38 had master’s degrees. Around 97 and 110 teachers teach at the junior and senior high-school levels, respectively. After observing the baseline data of respondents it was discovered that those who filled out the questionnaires were appropriate for the sample of mathematics teachers expected to achieve the study objectives.

### 3.3. Data Analysis

The variance-based structural equations modeling (PLS-SEM) technique was used for measurement and structural model analysis. Furthermore, the data testing steps suggested by Hair [99] were adopted. The PLS-SEM technique was considered the best for analyzing the relationship between variables and for observing the direct and indirect effects on each predictor with a small sample, without thinking about the data normality problem [100,101]. The PLS-SEM’s advantage was that it is suitable for testing new measurement models with more flexible variable numbers [102]. Finally, the use of smart PLS software for data testing was friendly for beginners and produced significant as well as professional results. According to Hair [103], two testing stages were conducted during the data analysis using smart PLS, the first is testing the measurement model, while the second is that of the theoretical model. Additionally, to analyze the differences in each variable based on gender and age, a t-test and one-way analysis of variance (ANOVA) were performed using SPSS software.

## 4. Results

It is important to reiterate that the PLS-SEM technique was used to examine mathematics teachers’ stress levels and well-being factors. The results were categorized into three sections, namely descriptive statistics and a measurement model, convergent analysis, and discriminant validity. Furthermore, the structural equations modeling was analyzed to conclude that this statistical method is consistent with the suggestion of Hair [103].

Table 2 shows the descriptive statistics of all measurement items. The average respondents answered more than 3.0, with a fairly low standard deviation of less than 1.0, meaning their answers were focused on the mean point. Skewness and kurtosis were expected to be between −3 and 3 to indicate that the data were normally distributed [104]. The kurtosis value ranged from −0.569 to 0.770, while the skewness was between −0.733 and 0.522. Consequently, it was concluded that the data were normally distributed.

### 4.1. Analysis Measurement Model

The outer loading value was initially considered to analyze the relationship between the latent variables and the outer model. Based on some references, the outer loading value is not supposed to be below 0.6, and preferably higher than 0.70 [103]. In this study, the value for all measurement items had the lowest value of 0.743 (WELL-BEING3), with the highest being 0.875 (TPMK2). Consequently, the data demonstrated a reliable item, and the details of the outer loading value having 24 measurement items are shown in Table 3. 

### 4.2. Convergent Validity

Convergent validity is used to measure the extent to which an item in a construct has a correlation or similarity. In addition, it is measured by each construct’s AVE value, which has to be more than 0.5 [103].

Another assessment used to analyze the outer model was construct validity. It determines whether the measurement item is capable to represent and analyze what is being searched. Based on Table 3, the lowest AVE value was 0.622, while the highest was 0.688. This means the first requirement to achieve good convergent validity was fulfilled. The next stage was to analyze the composite reliability value for each construct. As reported by Hair [103], the composite reliability value should not be less than 0.60. In this current study, it was greater than 0.80, and therefore, the entire construct was acceptable.

The value of Cronbach’s alpha was analyzed to assess each construct’s internal consistency. Internal consistency can be met when Cronbach’s alpha value is more than 0.70 [103], which is the limit commonly used in the social sciences. However, when a construct does not meet these criteria, then the convergent validity cannot be met, indicating that the construct cannot be continued to the structural model analysis stage. Table 3 shows that Cronbach’s alpha value was above 0.70, and hence the second stage of convergent validity was satisfactory.

### 4.3. Discriminant Validity

Discriminant validity is needed when predicting the dependent variable to assess the difference between one latent variable and another. A popular method for measuring discriminant validity as suggested by Hair [103] is the analysis of the correlation matrix among constructs. Specifically, the AVE value in each latent construct, as bolded in Table 4, is expected to be greater than the highest squared correlation of another latent construct. In this current study, all latent variables met the requirements.

### 4.4. Model Fit Measurement

A model fit in PLS-SEM was implemented conventionally to measure the estimated value and other latent constructs. The PLS-SEM approach using smart PLS software is capable of measuring the model fit by observing the unweighted least squares discrepancy (d_ULS) values, the geodesic discrepancy (d_G), and the standardized root mean squared residual (SRMR) [24,106]. It is important to mention that the fit of the model is usually considered good when the SRMR value is below 0.08 and the unweighted least squares discrepancy estimated is greater than the saturated model. Additionally, the geodesic discrepancy value in the estimated model is expected to be greater than that of the saturated model. The Normed Fit Index (NFI) value is considered as good when it is above 0.90 but still acceptable when it exceeds 0.60. Based on Table 5, all the requirements of the model fit suggested by Henseler [107] were met. Another method for assessing the model fit was analyzing the value of the root mean square error correlation (RMStheta). According to Henseler’s recommendation, the RMStheta should be less than 0.12. In this current study, 0.11 was achieved, which indicated a good model fit.

### 4.5. Structural Model Analysis and Hypothesis Testing

**Figure 2:**
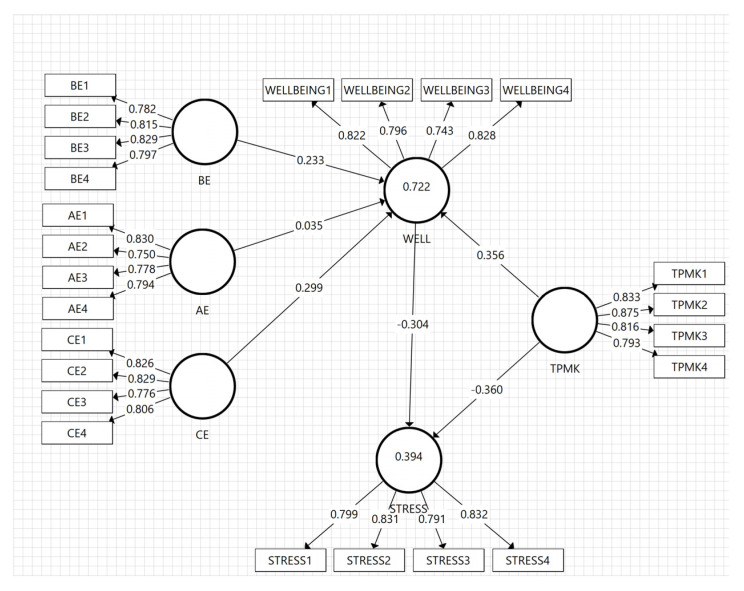
The outer loadings and R^2^ values. Note: AE: affective engagement, BE: behavior engagement, CE: cognitive engagement, WELL: well-being, and TPMK: Technological Pedagogical Mathematical Knowledge.

Structural analysis of the model begins with testing the determination coefficient, which is denoted by R2. It is usually used to indicate the extent to which the developed exogenous variables can explain the endogenous variables. In this study, the endogenous variables include the mathematics teachers’ stress levels and well-being. According to Hair [99], the minimum value for the variance was 0.10. Meanwhile, mathematics teachers’ stress levels and well-being were relatively high, with values of 0.394 and 0.722, respectively. This was interpreted that 72.2% of well-being was affected by behavior, affective, and cognitive engagements, as well as TPMK, while 27.8% was described by other factors not examined in this current study. The five predictors explained the teachers’ stress levels up to 39.4%, while 60.6% were affected by other factors. Figure 2 shows the details of the outer loading value and variance explained. The utilized model is valid and can more accurately measure the path significance related to these variables.

The next stage is testing the direct relationship between endogenous and exogenous variables by observing the path coefficients in the structural model. It was found that behavioral and cognitive engagement had a direct, significantly positive effect on mathematics teachers’ well-being (β = 0.233, t = 3.311, *p* < 0.01; β = 0.299, t = 40115, *p* < 0.001). However, effective engagement was not associated with well-being (β = 0035, t = 0.430, *p* > 0.05). Based on the results, cognitive and behavioral engagements significantly affected subjective well-being, while affective engagement did not. The feeling of happiness when teaching mathematics and meeting students is less indicative of the teachers’ subjective well-being.

Furthermore, an interesting and valuable result of this study is that TPMK was the most influential factor in reducing mathematics teachers’ stress levels (β = −0.360, t = 3.648, *p* < 0.001) and increasing their subjective well-being (β = 0.356, t = 4.745, *p* < 0.001). It was discovered that the ability to integrate technology into teaching students was the main factor related to the teacher’s well-being. In other words, when their TPMK is high, the stress levels tend to be low, thereby improving the teachers’ well-being. This is similar to the results of Luik [82], that TPACK directly affected teachers’ teaching abilities and reduces their stress.

**Figure 3:**
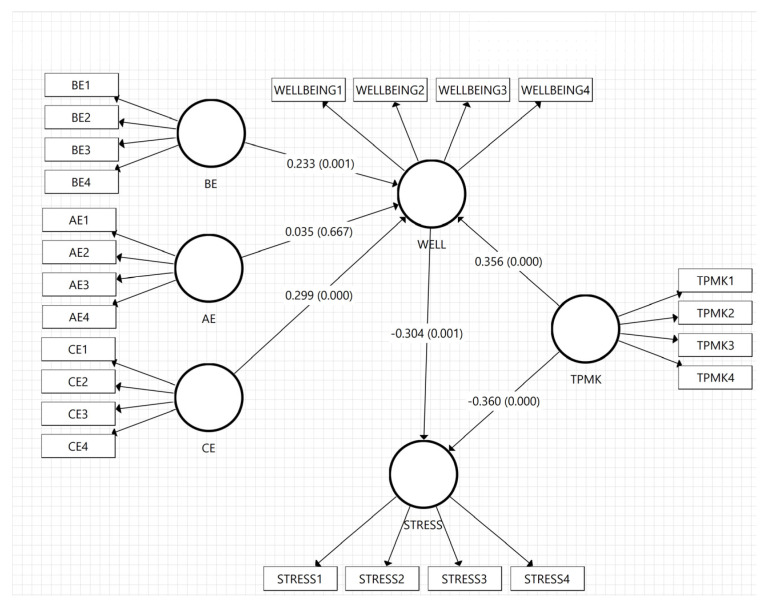
Discovery of structural model analysis with *p*-value and T statistics. Note: AE: affective engagement, BE: behavior engagement, CE: cognitive engagement, WELL: well-being, and TPMK: Technological Pedagogical Mathematical Knowledge.

Finally, well-being significantly reduced stress levels (β = −0.304, t = 3.289, *p* < 0.01). This means that the increase in well-being tends to reduce mathematics teachers’ stress levels. The stress levels are reducible by maintaining and improving the mathematics teachers’ well-being at secondary schools. This is consistent with several findings stating that there is a strong relationship between stress levels and well-being. However, subjective well-being is not a dominant factor in reducing stress levels. Table 6 and Figure 3 show the hypothesis testing, the T value, and the significance of the *p*-value.

### 4.6. Significant Differences Based on Gender and Age

This study examined the age and gender differences in mathematics teachers’ perceptions of engagement, stress, well-being, and TPMK levels. The t-test result in Table 7 showed a difference between males and females in the stress level (*p* = 0.005), but there was no significance on the other five variables. Furthermore, Table 8 describes the analysis result of the age factor on the engagement level, stress, well-being, and TPMK. It was observed that age significantly influenced the stress level (*p* = 0.022) but had no significant impact on other factors. It was found that teachers in the 21–30 age group had higher stress level than others (mean = 2.833).

## 5. Discussion

Stress levels often decrease teachers’ performance while teaching, and once left unchecked, can affect sustainable education [27,31]. Schools, where teachers work, ought to increase subjective well-being alongside reducing and managing stress levels caused by many factors, such as engagement [27]. Therefore, their job becomes more fun and innovation can be carried out with learning media or models for better performance as 21st century mathematics teachers [108,109]. This study aimed to: (1) determine the factors related to mathematics teachers’ stress levels and subjective well-being, and (2) observe those with the greatest significant effect on improving well-being and reducing stress levels. Based on the previous assumption, mathematics teachers’ stress levels are related to their TPMK and engagement.

Regarding cognitive engagement, the main focus was the mathematics teachers’ investment level in developing and innovating new models, as well as the one of effective learning approaches for improving their performance while enhancing students’ outcomes. When individuals’ cognitive engagement is high, their ability is often recognized by the people around them, thereby causing subjective well-being to increase [64,68,110]. In this context, teachers with good cognitive engagement can properly prepare their teaching materials. In addition, they are very committed to continuous learning to improve their pedagogical skills. For example, the teachers learn to utilize technology-based learning activities preferred by the students to help them understand mathematical concepts and improve their scores [111].

The final element in engagement theory is behavior, which is interpreted as an individual’s attitude toward an object [62,112]. Furthermore, engagement affects the people participating in activities and their achievements [67,112]. In this scenario, behavior engagement is the mathematics teachers’ attitude towards their profession and responsibilities. Teachers’ attitude is expected to continuously improve teaching skills and innovate with learning media according to the abilities and situations of students. It is safe to conclude that teachers with good behavioral engagement can motivate and increase their subjective well-being.

It has been previously predicted that affective engagement is related to well-being and stress levels [63,113]. Individuals with good affective engagement are usually able to communicate and motivate or positively affect people [63]. Furthermore, teachers with this kind of attitude can make the classroom atmosphere comfortable and know the students better. They tend to be happy with every class session, considering that the subject and students are part of their lives. These conditions may help to reduce stress levels and increase subjective well-being. However, mathematics teachers from a secondary school in Guangdong stated that affective engagement is not a significant factor in reducing stress levels. This showed that the location has fierce competition, and teachers’ qualifications are regularly reviewed, thereby causing cognitive and behavioral engagements to have more of an effect on their subjective well-being.

This study found that pedagogical mathematical knowledge of technology was the most important factor in reducing teachers’ stress levels and increasing their subjective well-being. This may indicate that teachers with good TPMK can be more contemporary while improving their teaching skills. For example, they can easily organize classes and adapt to students with different abilities in terms of low-stress levels. Additionally, ICT-based learning media can be used in explaining difficult mathematics material for easier understanding [114,115], and to obtain a sense of their well-being as teachers.

Finally, it was discovered that teachers’ well-being significantly reduced stress levels. Individuals with a high level of well-being generally have self-confidence, can motivate others, and can solve daily problems to reduce stress levels [68,116,117]. In this context, teachers with high well-being are not likely to be burdened with their responsibilities and duties. This may be because they rarely complain and do not feel their work is a burden. It is concluded that when the subjective well-being level is high, the stress levels tend to be low.

In addition to predicting the factors influencing teachers’ stress levels and well-being, this study further analyzed gender and age differences across all constructs. The results indicated that there were gender differences in stress levels, with male teachers experiencing higher levels than female. This is because all extracurricular activities and important tasks at school are often left to male teachers, thereby making their responsibilities heavier. At the same time, there is no difference in salary between both genders. This is the reason male teachers in Guangdong, China, have higher stress levels than females. Meanwhile, this result differed from the previous studies showing no difference in stress levels between both genders [86]. Other studies reported different results, where females had higher stress levels than males [118,119,120]. Consequently, the Chinese government has organized a program to improve teachers’ teaching skills, training in using technology-based learning media, and many administrative tasks.

There are also differences found in stress levels regarding the age factor. This is supported by previous studies indicating that age strongly impacts teachers’ perspectives [121,122]. It was discovered that young teachers between the age of 21 and 30 years have higher stress levels than those above 31 years. This condition is because young teachers were still adapting to the environment and teaching methods. Additionally, they are not experienced in effectively teaching mathematics, dealing with differences in student abilities in the classroom, and the various administrations of mathematics teachers in schools.

## 6. Theoretical Implications

The theoretical implication of this study was to build and examine a new model that can be used to predict mathematics teachers’ stress levels and well-being. From the observations, only cognitive and behavioral engagements had significance, while affective engagement produced no influence on well-being and stress levels. This study differs from others in that it focused on developing a model using engagement theory. Additionally, it concentrated on secondary school mathematics teachers, unlike others with a broader scope.

It was also discovered that TPMK was the strongest predictor of mathematics teachers’ well-being and stress levels. This understanding provided an opportunity to improve teachers’ well-being as well as students’ performance to realize sustainable goals.

## 7. Practical Implications

Practically, the results offered several recommendations for schools and teachers, as follows.

Schools need to be more attentive to mathematics teachers’ sustainable well-being rather than just focusing on their performance. When the teachers’ sense of professional identity and well-being increases, they tend to work efficiently [25,123]. At the same time, they are likely to feel confident and positive about being mathematics teachers, which benefits the school in the long term.

Schools are also expected to pay attention to mathematics teachers’ workload and normalize their stress levels. Teachers with behavioral engagement often make their classes more interesting and thereby become good to the students. This simply means that by moderately reducing work stress, teachers are more motivated to work by maintaining positive emotions.

Adequate technical support needs to be provided for mathematics teachers. Sometimes, teachers face practical obstacles in using certain technology despite the various tools available. Those capable of using different teaching techniques and media in delivering material tend to be more respected by students, thereby increasing their sense of well-being and stress. Therefore, schools can be better when adequate technical support is provided for mathematics teachers through funding or having the appropriate technical expertise.

This study further provided several practical implications for teachers. For example, they need to: (1) continuously improve technical knowledge and expertise in mathematics, (2) enrich the knowledge of mathematics and instructional technology, and (3) utilize different skills, such as communication, collaboration, and presentation, to realize different goals. Consequently, they can easily manage the classroom, gain a sustainable sense of well-being, and reduce stress levels.

Teachers’ engagement can be increased by attending training and discussing with others. Mathematics teachers are expected to properly manage their time and prepare teaching materials. This helps teaching and learning activities to run smoothly and indirectly reduces stress caused by unplanned activities. In addition, developing intentions and attitudes that are not easily discouraged often provides positive energy to improve well-being and reduce stress.

## 8. Conclusions

Teachers’ mental health and well-being in schools are mentioned in sustainable development goal 3. In general, well-being and stress levels are important factors affecting teachers’ performance while teaching. This study combined engagement theory and TPMK to form a new model with six hypotheses. The results showed that not all engagements significantly affected well-being and stress levels. Specifically, affective engagement had no significant effect, while cognitive and behavioral engagements produced a positive influence. The most important factor for increasing well-being and reducing stress levels was the TPMK. In conclusion, well-being was proven to reduce stress levels in mathematics teachers.

## 9. Limitations and Recommendations for Future Study

This study successfully validated a new model for reducing stress levels and improving well-being. However, some limitations need to be considered. First, the sample only involved 200 mathematics teachers in Guangdong Province, China, which is considered extremely small. Additionally, no further investigations or interviews were conducted to obtain more detailed results. This indicates that further study is needed to re-evaluate the developed model. A qualitative approach can be used to interview mathematics teachers at secondary schools about the factors affecting their well-being and stress levels.

The second limitation is that this study only developed a model based on Fredricks’ engagement theory. Several factors that change teachers’ behavior based on the system or environment often affect individuals’ engagement. Therefore, the relationship between engagement and sustainable well-being needs to be observed and reconfirmed to ensure its accuracy.

Finally, many predictors can still be developed to explain the factors affecting teachers’ stress levels. This simply means that further studies need to develop better models and measure the differences between the direct and indirect effects of demographic respondents on each variable.

## Figures and Tables

**Table 1:** The initial hypotheses of factors associated with well-being and mathematics teachers’ stress levels.

Hypotheses	Description
H1	Affective engagement has a positive effect on well-being
H2	Behavior engagement has a positive effect on well-being
H3	Cognitive engagement has a positive effect on well-being
H4	TPMK has a positive effect on well-being
H5	TPMK has a positive effect on mathematics teachers’ stress levels
H6	Well-being has a negative effect on mathematics teachers’ stress level

**Table 2:** Mean, SD, skewness, and kurtosis.

Measurement Items		Mean	Standard Deviation	Excess Kurtosis	Skewness
Behavior engagement	BE1	3.662	0.841	−0.569	−0.127
	BE2	3.657	0.812	−0.444	−0.164
	BE3	3.739	0.828	0.734	−0.611
	BE4	3.681	0.882	0.770	−0.733
Affective engagement	AE1	3.705	0.843	−0.415	−0.323
	AE2	3.681	0.801	−0.374	−0.209
	AE3	3.618	0.892	−0.241	−0.320
	AE4	3.647	0.956	−0.391	−0.310
Cognitive engagement	CE1	3.734	0.800	0.123	−0.396
	CE2	3.652	0.813	0.687	−0.582
	CE3	3.671	0.856	−0.273	−0.289
	CE4	3.657	0.903	0.442	−0.576
TPMK	TPMK1	3.676	0.861	−0.515	−0.283
	TPMK2	3.628	0.880	−0.601	−0.267
	TPMK3	3.725	0.849	−0.137	−0.392
	TPMK4	3.686	0.859	−0.384	−0.176
Subjective well-being	WELL-BEING1	3.710	0.859	−0.390	−0.186
	WELL-BEING2	3.710	0.870	−0.090	−0.331
	WELL-BEING3	3.696	0.868	−0.061	−0.349
	WELL-BEING4	3.633	0.912	0.084	−0.438
Mathematics teachers’ stress levels	STRESS1	2.498	0.962	−0.255	0.367
	STRESS2	2.391	0.936	−0.094	0.426
	STRESS3	2.425	0.897	−0.104	0.348
	STRESS4	2.329	0.942	0.143	0.522

Note: TPMK: Technological Pedagogical Mathematical Knowledge.

**Table 3:** Factor loading, reliability, and validity.

		Loadings	Cronbach’s Alpha	Composite Reliability	Average Variance Extracted (AVE)
Affective engagement	AE1	0.830	0.797	0.868	0.622
	AE2	0.750			
	AE3	0.778			
	AE4	0.794			
Behavior engagement	BE1	0.782	0.820	0.881	0.649
	BE2	0.815			
	BE3	0.829			
	BE4	0.797			
Cognitive engagement	CE1	0.826	0.825	0.884	0.655
	CE2	0.829			
	CE3	0.776			
	CE4	0.806			
Stress	STRESS1	0.799	0.830	0.887	0.662
	STRESS2	0.831			
	STRESS3	0.791			
	STRESS4	0.832			
TPMK	TPMK1	0.833	0.849	0.898	0.688
	TPMK2	0.875			
	TPMK3	0.816			
	TPMK4	0.793			
Well-being	WELL1	0.822	0.809	0.875	0.637
	WELL2	0.796			
	WELL3	0.743			
	WELL4	0.828			

Note: AE: affective engagement, BE: behavior engagement, CE: cognitive engagement, WELL: well-being, and TPMK: Technological Pedagogical Mathematical Knowledge.

**Table 4:** Fornell–Lacker analysis [105].

	AE	BE	CE	STRESS	TPMK	WELL
AE	**0.789**					
BE	0.792	**0.806**				
CE	0.819	0.802	**0.809**			
STRESS	−0.569	−0.582	−0.585	**0.814**		
TPMK	0.782	0.773	0.749	−0.599	**0.830**	
WELL	0.744	0.777	0.782	−0.587	0.788	**0.798**

Note: AE: affective engagement, BE: behavior engagement, CE: cognitive engagement, WELL: well-being, and TPMK: Technological Pedagogical Mathematical Knowledge.

**Table 5:** Model fit.

	Saturated Model	Estimated Model
SRMR	0.055	0.057
d_ULS	0.900	0.989
d_G	0.448	0.454
NFI	0.930	0.929
(RMStheta)	0.11	

**Table 6:** Results of initial hypothesis testing.

Hypothesis		β	Mean (M)	SD	T Value	Significance (*p* < 0.05)	Interpretation
H1	AE → WELL	0.035	0.039	0.081	0.430	0.667	Rejected
H2	BE → WELL	0.233	0.236	0.070	3.311	0.001	accepted
H3	CE → WELL	0.299	0.295	0.073	4.115	0.000	accepted
H4	TPMK → WELL	0.356	0.356	0.075	4.745	0.000	accepted
H5	TPMK → STRESS	−0.360	−0.355	0.099	3.648	0.000	accepted
H6	WELL → STRESS	−0.304	−0.317	0.092	3.289	0.001	accepted

Note: AE: affective engagement, BE: behavior engagement, CE: cognitive engagement, WELL: well-being, and TPMK: Technological Pedagogical Mathematical Knowledge.

**Table 7:** The t-test results of the gender factor on the engagement level, stress, well-being, and TPMK.

Variable	F	*p*-Value	Sig. (*p* < 0.05)
BE	5.181	0.240	No
AE	1.042	0.308	No
CE	2.927	0.089	No
TPMK	0.857	0.356	No
STRESS	8.004	0.005	Yes
WELL	0.394	0.555	No

Note: AE: affective engagement, BE: behavior engagement, CE: cognitive engagement, WELL: well-being, and TPMK: Technological Pedagogical Mathematical Knowledge.

**Table 8:** Analysis of age differences on the engagement level, stress, well-being, and TPMK.

Variable	F	*p*-Value	Sig. (*p* < 0.05)
BE	0.325	0.808	No
AE	0.402	0.752	No
CE	0.187	0.756	No
TPMK	0.118	0.877	No
STRESS	1.850	0.022	Yes
WELL	0.133	0.849	No

Note: AE: affective engagement, BE: behavior engagement, CE: cognitive engagement, WELL: well-being, and TPMK: Technological Pedagogical Mathematical Knowledge.

## Data Availability

Not applicable.
